# Understanding the roles of three academic communities in a prospective learning health ecosystem for diagnostic excellence

**DOI:** 10.1002/lrh2.10204

**Published:** 2019-12-02

**Authors:** Katherine Satterfield, Joshua C. Rubin, Daniel Yang, Charles P. Friedman

**Affiliations:** ^1^ Department of Learning Health Sciences University of Michigan Medical School Ann Arbor Michigan; ^2^ The Gordon and Betty Moore Foundation Palo Alto California

**Keywords:** diagnostic error, learning community, learning health systems, machine learning, medical diagnosis, multidisciplinary

## Abstract

Inaccurate, untimely, and miscommunicated medical diagnoses represent a wicked problem requiring comprehensive and coordinated approaches, such as those demonstrated in the characteristics of learning health systems (LHSs). To appreciate a vision for how LHS methods can optimize processes and outcomes in medical diagnosis (diagnostic excellence), we interviewed 32 individuals with relevant expertise: 18 who have studied diagnostic processes using traditional behavioral science and health services research methods, six focused on machine learning (ML) and artificial intelligence (AI) approaches, and eight multidisciplinary researchers experienced in advocating for and incorporating LHS methods, ie, scalable continuous learning in health care. We report on barriers and facilitators, identified by these subjects, to applying their methods toward optimizing medical diagnosis. We then employ their insights to envision the emergence of a learning ecosystem that leverages the tools of each of the three research groups to advance diagnostic excellence. We found that these communities represent a natural fit forward, in which together, they can better measure diagnostic processes and close the loop of putting insights into practice. Members of the three academic communities will need to network and bring in additional stakeholders before they can design and implement the necessary infrastructure that would support ongoing learning of diagnostic processes at an economy of scale and scope.

## 1 INTRODUCTION

In its breakthrough 2015 report, *Improving Diagnosis in Health Care*, the National Academy of Medicine (NAM) argued for new approaches for health care organizations (HCOs) to “identify, learn from, and reduce diagnostic errors and near misses in clinical practice.”^1^ This is but one of several publications documenting the extent of missed, delayed, and miscommunicated diagnosis as a cause of unnecessary morbidity and mortality.^2^, ^3^, ^4^, ^5^, ^6^, ^7^ However, because diagnostic processes are difficult to objectively measure, diagnostic quality and safety efforts have largely been eclipsed within the patient safety movement by treatment errors.^8^ As such, diagnosis has assumed the status of a systemic and persistent “wicked problem” for health and health care.^9^

Over the past decade, since the introduction of the concept by the (then) US Institute of Medicine,^10^ the learning health system (LHS) has emerged as a method for addressing large‐scale health challenges.^11^ The potential of applying LHS methods to address the wicked problem of diagnostic error motivated program officials of the Gordon and Betty Moore Foundation to suggest an exploratory study of how this connection might be achieved and, more specifically, how the characteristics of a functioning LHS^11^ can support the cultural and technical changes required to pursue diagnostic excellence. In particular—through the mandate to learn from every patient and their health experiences^12^, ^13^ and through the infrastructure‐supported ability to rapidly deliver new knowledge into practice^12^—there are sound reasons to believe that LHS approaches hold the potential to catalyze efforts toward accurate and timely diagnoses.

The NAM report on diagnostic error outlines broad goals to address the infrastructural, procedural, and cultural pitfalls that are perpetuating these errors in health care.^1^ In doing so, it lightly alludes to the coordinated, system‐based approach that LHSs can offer such a deeply systemic problem.^14^

With support from the Gordon and Betty Moore Foundation, we explored the collaborative potential to apply LHS approaches toward diagnostic “excellence”: a term that goes beyond reducing error and encompasses optimizing the often competing factors of timeliness, cost, and patient experience. Resting on the precept that multistakeholder learning communities are fundamental to the LHS concept, we explored how three distinct academic disciplines, guided by differing sets of underpinning sciences and associated methods, might interact to further the promotion of diagnostic excellence. How have (or have not) these communities interacted thus far, and how can weaving them together create a stronger approach to this wicked problem?

The first community comprises researchers and advocates for improving diagnosis (IDx), a group that largely utilizes methods associated with clinical research informed by behavioral and cognitive sciences. Concerns with diagnostic error and delay have gradually garnered the attention of researchers, beginning with (though somewhat overshadowed by) patient safety movements in the mid‐1990s.^2^, ^4^, ^8^, ^15^, ^16^ Toward the end of the first decade of the 21st century, leading researchers in diagnostic error began to form a more organized coalition, resulting in the formation of the Society to Improve Diagnosis in Medicine (SIDM). SIDM has successfully advocated for greater attention to diagnostic errors, in particular by charging the NAM to create the *Improving Diagnosis in Health Care* report.^1^ Concurrently, diagnostic researchers have focused heavily on defining the size and scope of the diagnostic error problem,^2^, ^3^, ^17^ creating taxonomies of cognitive and systemic causes,^2^, ^18^, ^19^, ^20^, ^21^ and testing interventions for identifying and reducing specific types of errors.^22^, ^23^, ^24^, ^25^ Supported by this forward momentum, it appears that the IDx movement is poised to make a large impact if systemic, coordinated infrastructure with broad utility for quality improvement delivery is put in place.

Our second community of focus concerns researchers in machine learning and artificial intelligence (ML/AI) in health care. These computer scientists, in collaboration with clinicians, use complex medical data—including from electronic health records (EHRs), medical imagery, sensors, and genomes—to create inferences that can be used to improve care. The Moore Foundation charged us with exploring how these tools might catalyze diagnostic improvements—a supplemental, 21st century counterweight to the cognitive biases that occur during diagnosis. Recently, ML/AI researchers have made headlines with tools that can diagnose certain conditions with higher positive predictive values than the average clinician.^26^, ^27^, ^28^ However, although the appropriate use of AI in health care is frequently debated within medical literature,^29^, ^30^, ^31^, ^32^, ^33^, ^34^, ^35^ there is little published attention to how any given tool can be used in the clinic. It is clear that the ML/AI community has a place in pursuing diagnostic excellence, but the exact dynamic of this intersection remains unclear. What is certain is that this goal requires ML/AI stakeholders who understand the dynamics of collaborating with physicians and other clinical experts.

The third community encompasses LHS researchers and implementers, a group comprising a mélange of biomedical informatics and system, policy, and implementation sciences. Members of this group use their diverse backgrounds and experiences to advocate for health system reform that incorporates scalable continuous learning that can be applied to any health problem. We hoped to leverage their expertise to illustrate how to improve diagnostic outcomes at broader scale and scope than could previously be completed. Furthermore, we proposed that the LHS methods employed by this community could provide a framework for the intersection of the IDx and ML/AI communities. We ask, how can systems better link knowledge generated by ML/AI researchers and the much needed changes to clinical practice, championed by the IDx movement?

This paper reports the initial step in generating a mature vision of an LHS approach toward diagnostic excellence endorsed by diverse stakeholders. We designed the project in two phases. The first phase, reported here, explored the interests and perspectives of three contributing research communities: members of the IDx community, researchers applying ML/AI to health care, and LHS researchers. We accomplished this via 32 key‐informant interviews, designed to capture the landscape of the three research communities and insights into their potential for interaction. The second phase, the results of which will be described in a separate publication, convened multidisciplinary collaborators at a 2‐day meeting.

## 2 RESEARCH QUESTIONS

We designed our research questions to inform a meeting of all three research communities around the aim of diagnostic excellence. To fully envision how all three communities can contribute to diagnostic excellence, we broke our aim into three questions that each provide valuable context to understanding a potential collaboration. First, we sought to understand the landscape of each community by defining both its history and trajectory toward collective goals, as well as the barriers and facilitators to meeting those goals. Second, we contextualized the activities of each community as they relate to the LHS construct that new knowledge can and should be rapidly translated into practice, asking how each community approaches, promotes, or facilitates translation and dissemination of findings into clinical practice. Finally, so that we could be aware of and account for the interdisciplinary dynamics, we needed to discern each community's level of understanding of and attitudes toward the two other fields in this potential collaboration.

## 3 METHOD

### 3.1 Key‐informant selection

Our key‐informant interviews comprised 32 experts dedicated to improving diagnosis (n = 18), ML/AI in health care (n = 6), and LHSs (n = 8). Informants were contacted by the principal investigator (C.P.F.) via e‐mail explaining the project and inviting them to schedule an interview via phone or in‐person at the 2017 Diagnostic Error in Medicine (DEM) Conference in Boston, MA. We identified informants from each community as follows.

#### 3.1.1 Diagnostic improvement

We contacted the primary investigator from each grant in the Gordon and Betty Moore Foundation's Diagnostic Excellence investigative program (n = 9). Additionally, we spoke with a leader in SIDM to expand our list to include other key researchers and patient advocates (n = 9). All 18 informants agreed to be interviewed about their field, seven of whom were interviewed in‐person at DEM.

#### 3.1.2 Machine learning and artificial intelligence in health care

To identify ML/AI researchers who might be interested in this project, we connected with a colleague who is a leader in the Machine Learning for Healthcare conference group. We reviewed titles and abstracts presented at their 2017 meeting to create a shortlist of researchers who might be interested in ML in ambulatory diagnosis and/or demonstrate interdisciplinary collaboration. We contacted nine researchers, six of whom responded to the invitation and then scheduled an interview.

#### 3.1.3 Learning health systems

We drew on our extensive network of LHSs professionals to identify a list (n = 8) of researchers and advocates who focus on a broad array of approaches and concerns in developing LHSs. All of the identified individuals agreed to the interview.

### 3.2 Interview procedure and guides

Interviews were semistructured, approximately 30 minutes in duration, and adaptable to the interviewees' insights and direction. The interviewer first offered a summary of the project and asked permission to record for note‐taking purposes.

We created a separate interview guide for each community of study (full interview guides can be found in the Supporting Information). Interview guides began with an in‐depth focus on the interviewees' own discipline: learning about their research journeys as a proxy for the history and trajectory of their fields. We generally surveyed their discipline's significant accomplishments, important questions, and major needs or barriers.

Later questions addressed our second and third research questions, inviting thoughts on the potential for collaboration among these three research communities. Through this stage of the interview, we gauged familiarity with and attitudes toward LHS concepts and how they could relate to diagnosis. For example, we asked how each discipline approaches translating knowledge into practice and implementing lessons learned. Additionally, we prompted interviewees to respond to the idea of using “big data” and/or ML to assist with problems related to medical diagnosis. These open‐ended questions were designed to spur insights into the dynamics at play before we planned a convening of interested parties.

Because the interviewer was a representative of LHS researchers, special care was taken to demonstrate neutral positions, prevent leading questions on the functions and goals of LHSs (unless requested after LHS‐related prompts had been discussed), and invite open dialogue. Following the discussion, we encouraged interviewees to contact us with any further thoughts. All interviewees were invited to join us for the meeting in phase two, which was designed around the results from these interviews.

### 3.3 Analysis

Interviews were recorded concurrently with note‐taking in Microsoft OneNote, which enables timestamped playback for review of each new line of text. During the conversation, the interviewer grouped notes according to over‐arching concepts in the interview guide. These varied slightly for each of the three disciplines, but were roughly divided into groups representing the interviewee's discipline, followed by distinct sections for perceptions of and interactions with the other two research communities. After each encounter, the interviewer reviewed gaps in the notes, summarized information collected, and highlighted insights into interdisciplinary dynamics and insights into the fields, assisted by the timestamped recording.

Once collected data approached saturation and/or all scheduled interviews were complete, we initiated formal analysis of the complete interview notes from each discipline. We iteratively reviewed interview notes with an inductive approach to build a thematic analysis. Using the list‐making web‐application Trello, we organized and analyzed data into themes, which in turn were categorized into four domains that emerged as a high‐level synthesis of the questions asked of each individual community. The results are presented according to these domains: work on diagnostic excellence, ML/AI in health care and for diagnostic excellence, LHSs and their application for diagnostic excellence, and clinical translation. To assist interpretation of themes in the context of potential collaboration, all themes were also tagged to mark tensions, open questions, and the discipline(s) that addressed the theme during interviews. Finally, throughout interpretation, we were mindful of potential biases that may have occurred in the interviews because the interviewer was a representative of established LHS researchers.

## 4 RESULTS

### 4.1 General perspectives on pursuing diagnostic excellence

The IDx researchers are proud of the community they have established over the last decade and of subsequent achievements that have put diagnostic harm on the radar as a significant patient safety concern. Despite this growing momentum, interviewees in the ML/AI and LHS communities were relatively unfamiliar with the diagnostic improvement space, signaling the need for continued visibility of the IDx community and movement. In a similar vein, IDx interviewees generally agreed that because the diagnostic process is much less visible than medical treatment, diagnostic error must be addressed separately from traditional quality and safety approaches. Without this separation, they argued, it is likely that diagnostic error will continue to remain in the background of more highly visible, measurable opportunities to prevent iatrogenic harm and optimize therapeutic outcomes. This view is accompanied by a general sense that their community does not agree on the definition and scope of diagnostic error and have mixed approaches to propelling the movement forward. However, they agreed on a number of barriers that prevent the full integration and acceptance of diagnostic excellence as a critical component of safe and quality care.

#### 4.1.1 Data and measurement barriers to diagnostic excellence

IDx experts frequently emphasized the need to standardize and tighten the feedback loop that informs care providers of distal diagnostic outcomes of patients. As one interviewee quipped, “If an individual doctor makes a diagnosis and doesn't ever hear that the patient turned out to have a different diagnosis and got re‐admitted, or it turns out their diagnosis was wrong, then [the doctor] will never get better.” IDx participants commented on the inherent difficulty gathering these data if the correct diagnosis is made by another clinician or, even more so, in another health system. As a response, many seek ways to better engage the patient who has the power to “tell us if we got something right.”

This need for feedback is predicated on the need for measures that both define and track error. One interviewee recalled research that found that harm from diagnostic error is often identifiable among patient safety reports, but not consistently mapped to diagnostic error as the root cause. This issue compromises efforts to form HCOs' “business case” for creation and implementation of improved diagnostic measures. One participant called for public accountability efforts similar to those for patient safety, which might create the necessary pressure on HCOs. These interviews did not reveal deeper insights into how to gain commitment from HCOs, though barriers were largely presumed to be fiscal. However, one interviewee hypothesized that difficulty in funding research in diagnostic error may also be impeded by clinicians who review funding opportunities, as they may feel criticized by the notion that they and their peers are not diagnosing patients adequately.

#### 4.1.2 Behavioral barriers to diagnostic excellence

In that regard, many interviewees referenced various ways that clinicians may inadvertently impede diagnostic improvement. Experts cautioned that physicians do not have the tools to self‐assess diagnostic skills. One clinician spoke about approaching doctors to think about this problem: “A lot of them have their hackles go up when you start talking about error. They all know about it because they all know about malpractice and the risk of malpractice, but they all think it's somebody else that's not as good as them, or not as careful, or not as well trained.” The interviewees who spoke about this issue cited several drivers, rooting the cause in medical school, which they said often lacks adequately explicit training in the diagnostic process even while emphasizing the importance of clinical reasoning. Instead, diagnostic processes are treated as “private mental events” in the head of an expert (ie, physician) who does not need to document an explanation. Furthermore, the prevailing culture promotes the idea that doctors can and should memorize all medical knowledge, upholding a social norm that to need to reference secondary sources is a sign of incompetence. Interviewees argued that these cultural factors cause physicians to miss opportunities for patients to be better served with consults from other doctors or use of diagnostic support systems.

This barrier is perhaps exacerbated by suboptimal data from physical exams and patient history. The physicians we interviewed desire more comprehensive, longitudinal health data that would enable more rigorous interactions with relevant health history. Together with other behavioral changes, this could potentially improve bedside diagnostic processes, including history‐taking, physical exams, test selection and interpretation, and development of the differential diagnosis. However, current weaknesses are ambiguously attributable to both clinician procedure and EHR design that might inhibit accurate data recording.

### 4.2 General perspectives on machine learning/artificial intelligence in health care and its potential application toward diagnostic excellence

The six computer scientists we interviewed in the ML/AI community have recently applied a variety of methodologies toward improving health care delivery and administration. These approaches range in scope and area of effort. Some use natural language processing (NLP) to synthesize relevant information from journal articles and EHRs for a clinical team's immediate use. Others leverage predictive modelling and causal inference to make highly generalizable conclusions. Still others described how they can use control theory/Q‐learning/back propagation techniques to identify pivot points or errors in a clinical pathway. In all cases, their research interests are grounded in a desire to answer complex questions through data, and the health care domain poses unique challenges that influence their approach. They advocated collaborating directly with clinicians on questions of causality, rather than simply increasing the accuracy of a prediction. One ML/AI researcher pointedly observed, “I care more about action‐ability and lead time than positive predictive values.” This is echoed in a set of guiding characteristics that another researcher identified through direct work with clinicians: that models must be actionable, or have utility in practice; that they are robust as clinical conditions and context change; and that models should be made more credible by leveraging what is already known from the literature.

A critical component of integrating ML/AI methods with health care is establishing meaningful and mutually beneficial collaborations among researchers, clinicians, and others involved in the care process. As one researcher pointed out, “Because of the nature of the type of research we do … we have to have work used …. It is as hard as coming up with machine learning techniques.” This researcher stressed relationship building and sometimes doing “service” work with health systems contacts on questions that are less academically interesting in order to support this process. Other experts have sought collaborations in more integrated ways, such as by bringing physicians into their labs as colleagues and by training physicians as doctoral students in computer science. Overall, these approaches have helped computer scientists identify clinically relevant questions, conduct clinically accurate analyses, and obtain access to data. The latter has been historically challenging for computer scientists. “Shareable data,” one researcher said, “have been key to allowing researchers to really benchmark their data and risk‐stratification algorithms, which has led to significant advancements.” However, there are still difficulties implementing their results into clinical settings. A few researchers described licensing their algorithms to third‐party companies as an avenue to put these products into meaningful practice, though one recounted being rebuffed by a major producer of digital medical products that did not want to undertake the FDA approval process.

#### 4.2.1 Potential collaboration

Recognizing that this sample is limited to six ML/AI computer scientists and 18 diagnostic researchers, evidence emerged from these interviews that the ML/AI and IDx communities have not deeply interacted. Of particular importance, the computer scientists did not readily offer many examples of ML/AI work specific to medical diagnosis. Two of these researchers very vocally emphasized addressing treatment over diagnosis, believing that physicians are not interested in diagnostic support and that the potential impact is not worth the effort. In the words of one researcher, “Interesting questions lie in the iterative improvement of the treatment plan, rather than in the diagnostic process.”

On the other side of the collaboration, IDx researchers acknowledged the potential to apply “big data” and ML/AI techniques to their field, but also expressed significant apprehension. First and foremost, they were concerned about the quality of available data. As (many of them) physicians, they understood firsthand the unreliability of billing data and free text in the EHR, questioning the validity and utility of any conclusions made from those sources. Some expressed general skepticism about the value of these methods beyond existing achievements in imaging‐related fields such as radiology, dermatology, and pathology.

Many of the IDx researchers asserted that any ML algorithms that might be applied to health care must be designed to be understandable by the clinicians making care decisions. The underlying tension on this point was echoed by members of the other two communities. To one computer scientist, this means creating a dialogue to counter the “myth that AI algorithms are black boxes.” This was corroborated by an LHS interviewee with decades of work on diagnostic support tools, who emphasized that providing explanations for “black box” algorithms is critical to engendering trust in ML/AI methods. Other LHS researchers, however, proposed more broad solutions through building interactive, interpretable systems where knowledge is intentionally rendered in a form in which clinicians can review, edit, and disseminate it. Additionally, one IDx physician argued that the unknown consequences of AI in health care demand that stakeholders develop a certification process for algorithms' use in the health care setting. Regardless of the approach, it seems clear that application of ML and AI methods toward diagnostic excellence requires increased dialogue between interested parties. This dialogue would both create awareness of mutual interests and build consensus on the scope of this proposed intersection.

### 4.3 General perspectives on learning health systems and their potential application toward diagnostic excellence

In interviews with LHS experts, we found that it can be difficult to adequately discuss the potential for LHS‐based solutions when current examples are typically scaled to single health systems and not fully mature. At the same time, interviewees expressed belief that implementation of small‐scaled LHSs remain an important stepping stone toward interoperability on a much larger scale. For example, one expert spoke of securing buy‐in to LHS methods: “It's a concept, I know, that polarizes people. And what would really help in terms of moving this on is if we could at least in a couple of areas build up some genuine exemplars that have the potential to scale.” Building these exemplars would concretely demonstrate feasibility, generate data on the persistence and effects of the organizational learning that occurs, and attract more adopters of an LHS model.

Furthermore, LHS interviewees agreed that technical issues were not the major challenge to LHS development. A former computer scientist from the LHS community stated: “[emergence of a large scale LHS] was unlikely to be achievable using traditional software and systems engineering thinking and [their own ideas were] more focused on establishing conditions under which the right sorts of infrastructures and dynamics would emerge.” In other words, the development of larger scale systems is contingent on incentivizing cooperation between and within stakeholders in the health industry.

Similarly, a common theme between LHS interviews highlighted the necessity to form a sociotechnical system solution that links the efforts and interests of all stakeholders at multiple points in a care pathway to address system‐level changes and concerns. One LHS expert emphasized that future success is dependent on whether, “the culture of the care process is one in which decisions are made in a team fashion rather than in a linear, traditional, clinician‐to‐patient unidirectional fashion.”

#### 4.3.1 Potential collaboration

Overall, results of interviews with eight LHS experts reflected the interdisciplinary background of this diverse group of researchers and advocates, including former physicians, computer scientists, and individuals with expertise in health information technology, health policy, and population health. As such, their perspectives echoed many themes expressed by the IDx and ML/AI communities, incorporating calls for actionable algorithms, shared data, and clinician feedback. In general, they offered a visionary roadmap for systems changes, promoting the potential of LHS approaches to provide context in rapidly advancing health care. As one leader put it, “We could significantly tailor diagnostic tools more appropriately to the individual circumstances, and part of it will emerge through development of decision tools that will help individual clinicians contend with the fact that knowledge availability and knowledge needs far surpass the human capacity.”

When questioned about adapting LHS approaches to diagnostic problems of interest, one former physician‐turned LHS advocate expressed excitement over the untapped opportunity of accessing “breadcrumbs” in the EHRs for cracking diagnostic problems that run over long periods of time. Although this feasibility of using EHR data was common among all three communities, this proposal was relatively unique among the LHS group, in part for its tailoring to the needs of the diagnostic process. In contrast, a few LHS researchers pointed out that their field is domain‐agnostic and they dismissed potential disciplinary challenges in using LHS approaches for diagnosis. This tendency is visible across insights from the LHS interviews, which are frequently centered on LHS perspectives rather than anything specific to diagnosis. On further reflection, one interviewee acknowledged that “diagnosis is a process that perhaps has more uncertainty”—and more complexity on the knowledge‐to‐performance (K2P) side of the learning health cycle—compared with other LHS applications and earlier automated decision support systems (such as what is used for drugs). With this in mind, the researcher went on to describe the potential collaboration of these three communities as the “poster child” for how learning health infrastructures must complement existing human‐completed processes. In other words, full automation is not a viable option.

On that note, members of the IDx group generally supported the concept of LHSs for diagnostic excellence, particularly by endorsing organizational‐level learning and improving. Some ML/AI researchers optimistically viewed LHSs as an avenue to better incorporate patient histories and diversity into our standard of care (compared with the standard of learning from randomized clinical trials). Overall, responses from the two other domains were neutral or positive, though only a few IDx experts had a strong conceptual grasp of LHSs as cultural and infrastructural endeavors (rather than as single, isolated feedback loops). These interviewees could comfortably integrate their actionable agendas for diagnostic improvement into LHS scope and scale.

### 4.4 Clinical translation

Finally, responses to our questions about translating research into practice highlighted the potential benefit that LHS approaches can offer the diagnostic problem space. Expressing frustration with “all these papers and no uptake,” a few IDx interviewees discussed interest in wrapping multidisciplinary IDx researchers into an HCO to directly link research and clinical practice. However, as pointed out by some interviewees in multiple communities, there are high‐level policy‐level and cultural changes that would need enacted before this could become routine. “We need to develop a clear societal understanding,” an LHS researcher said, “that we expect systems to learn from practice they do and to share that info without running afoul of [the] oversight regime that covers research.” However, the future of how we distinguish traditional clinical research and iterative health system learning remains unclear, and these interviews did not offer a roadmap toward a solution.

## 5 DISCUSSION

### 5.1 Piecing together the collaboration—themes and insights from the interviews

**Figure 1:**
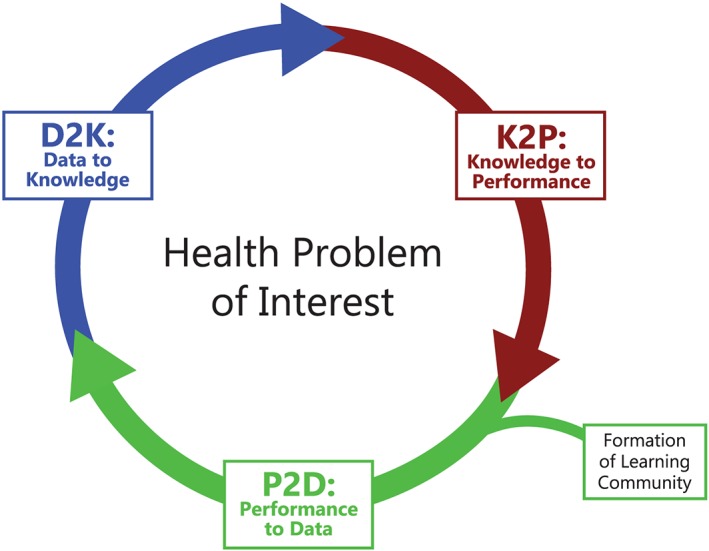
The Learning Cycle begins with the formation of a learning community that collects sufficient performance data to engender learning. Adapted from Friedman, Rubin, and Sullivan (11).

Many of the themes derived from the interviews illustrated that these three communities are compatible—if not ripe—for deeper collaboration. In reviewing the results of the interviews, we quickly realized a natural fit, in which the expertise of the three communities loosely correspond to a high‐level abstraction of an LHS: the learning cycle (depicted in Figure 1). The learning cycle is represented by three stages, initiated when a learning community organizes around a problem of interest. They collect performance and health outcomes as analyzable data (performance‐to‐data [P2D]); data are analyzed into new knowledge or insights about the problem or how to address it (data‐to‐knowledge [D2K]); knowledge is strategically directed into changes in health practice (K2P); and the cycle starts again at P2D.^11^

The IDx community has spent a few decades building strong research programs to further define the scope and causes of harms from diagnostic error. However, the experts we interviewed cited their persistent difficulty seeking to learn from every patient and health event. In particular, they desired data collection of distal patient outcomes, including indicators of diagnostic performance that are recorded by other physicians or other health systems. Although they have laid the building blocks for P2D, and continue to create standardized measures of diagnostic processes,^36^, ^37^, ^38^ partnering with LHS thinkers could potentially improve linkages across systems. This would also improve quantity and representation of currently collected measures.^39^ For instance, certain rare diseases or atypical presentations of common diseases may not have sufficient incidence (even in regional health networks) to supply sufficient statistical power to learn about and improve the rate of timely and accurate diagnosis.^24^ The solution to this problem—sharing large amounts of data across health systems—has the potential not only to increase power for a particular condition but also to increase power for understanding the effects of demographic and socio‐economic characteristics. To take a drug safety surveillance analogy, at a 2013 conference on data‐driven health care decision making, Dr Larry Norton noted that it took the FDA over 5 years to recognize that the drug Vioxx was causing a significant increase in fatal myocardial events and withdraw it from the market. In a health data‐sharing network comprising eight million patients, a safety signal could have been detected in under half that time. With 150 million patients represented, it would have taken under 6 months, and if the entire nation were sharing data, the signal would have been realized in under 10 weeks.^40^ Networked systems with data sharing are requisite to fully and rapidly learn from and for all patients.^30^, ^41^, ^42^

Furthermore, the IDx and ML/AI groups are critically disconnected, slowing the introduction of new analytic (ie, D2K) methods that might reveal diagnostic breakthroughs.^34^, ^39^ These innovative methods, especially when accompanied by new measurements of diagnostic process, offer more than high‐performing predictive diagnostic algorithms. They could also facilitate diagnostic excellence by providing insights into the balance between diagnostic accuracy, timeliness, cost, and patient experience. However, these aspirations cannot be realized without tightly‐knit collaborations between clinical stakeholders and the researchers who can build such tools. As we heard in interviews from all three communities, clinicians are generally not interested in tools that predict a diagnosis. To overcome cultural barriers, clinical and research stakeholders need to create sociotechnical diagnostic support processes that encourage and offer consultation. Although research on uptake is relatively slim,^43^ we can see this with the current generation of diagnostic support software, such as VisualDx and Isabel, which give clinicians tools to explore symptoms and probable diagnoses.

Finally, health systems are not designed in a way that properly promotes uptake of diagnostic best practices into clinical practice (ie, K2P). Although this barrier was not frequently stated in interviews with IDx experts, it is implicit in the historical difficulty in bringing attention to and improving diagnoses.^1^, ^8^ As our LHS interviewees commented, we are in desperate need of system‐based solutions that catalyze K2P, institutionalizing the pipeline—curation, management, and dissemination—that translates evidence‐based medicine and ML algorithms into the clinical exam room.^31^, ^44^

Perhaps most importantly, interviewees in all groups called for multistakeholder engagement. The involvement of stakeholders from clinical, computer science, and systems and implementation science disciplines—plus the critical voice of patients—will engender shared appreciation for the strengths and limitations of methods to create lasting changes in diagnostic quality.^32^, ^33^, ^36^ This collaboration also has the potential to build a solid LHS exemplar to further the vision for other health questions. Together, these groups can create and sustain the systemic solutions needed to create diagnostic excellence.

### 5.2 Limitations

The design of this project limited the scope and perspective of the results. Interviewees may have had social and professional incentives to speak favorably when discussing potential collaborations. More directly related to the prospects of a future LHS for diagnostic excellence, the scope of the project was limited to three broad domains that were commonly grounded in academic relationships. Any practical next steps would need to include stakeholders that were not specifically brought into this discussion, such as patients, caregivers, and their advocates; HCOs and the policy, administrative, and legal perspectives that support them; a range of clinical professionals involved in diagnostic processes; additional implementation science experts; and quality and safety organizations.

### 5.3 Steps toward a learning ecosystem for diagnostic excellence

The vast and complex scope of factors that contribute to diagnostic error^1^, ^9^, ^45^ inspires a vision of a learning ecosystem that evolves from existing (often local) projects into networked learning communities and learning cycles.^11^ Beginning with projects that address diagnostic problems of interest that cause the greatest harm,^46^ an emergent, ecosystem approach builds on existing momentum to develop diverse multistakeholder learning communities that will optimize diagnosis for patients and providers. With dedication to broad and deep stakeholder involvement, learning communities will be well equipped to govern learning cycles that are clinically meaningful, sensitive to patient needs, informed by good science from a variety of methodologies (including ML/AI in some cases), and safely implemented.^47^, ^48^

However, there are many steps to be taken before this vision can be actualized. First, the right stakeholders must be mobilized, and although our interviews show the potential for synergy, that conclusion needs to be validated by the members of the three communities. Our next step is to convene members of these three stakeholder communities. This convening will provide a space to network and prototype how to synergize these communities in an LHS around specific diagnostic problems. For example, it is an opportunity to build a relationship between the IDx and ML/AI communities by helping IDx researchers better understand the capacity of ML/AI work and by creating space for ML/AI researchers to learn more about the IDx need.

We view the LHS community's role as one that provides a contextual framework that can help facilitate data collection, clinical translation, and a community of practice that can navigate the cultural barriers that the IDx movement faces. A large portion of the role of LHS advocates is to promote the development and adoption of a complete platform of infrastructural services. A complete sociotechnical infrastructure—to be built of policies, processes, and technologies, carried out by people—is the enabling force behind an ecosystem's ability to grow in scope and scale. Existing example infrastructure components include D2K‐enabling platforms that manage and share clinical data, such as PopMedNet,^49^, ^50^, ^51^, ^52^ and “big data” platforms and algorithmic toolkits that are routinely used by the ML/AI community.^50^, ^52^, ^53^ There are also many emerging components that support K2P, such as the Knowledge Grid (KGrid)—which stores health knowledge and generates tailored advice to drive practice change^54^, ^55^—and standards that support interoperability of such services.^56^ On the other hand, there appears to be a great need for infrastructure that supports P2D. Diagnostic researchers are calling for better‐structured reporting of diagnostic processes and outcomes that can support our understanding of the epidemiology of diagnostic errors and generate new answers to addressing these challenges.^18^, ^45^, ^57^

Another major stakeholder component includes patients and patient advocacy and safety groups. These groups are well positioned to be particularly effective in building a business case—or other, policy‐based incentives—for HCOs and insurers to support this work. For example, can these groups work with payers to advocate for improved timeliness and communication? Could they support research that studies the outcomes of diagnostic excellence efforts to help incentivize HCOs? Momentum around particular causes of diagnostic harm, and the motivation to address specific areas of diagnostic scope, is a crucial first step to activating stakeholders of medical diagnosis.

It has been said that if one has 1 hour to save the world, the first 55 minutes should be spent defining the problem. Diagnosis is a critical point in the treatment path, and it follows that systems should assess diagnostic processes for opportunities to optimize patient outcomes. Yet, for reasons that were illuminated in our interviews, cutting edge innovations in ML/AI and process improvements anchored in LHS approaches have disproportionately focused on treatment and quality, without corresponding investments toward realizing diagnostic excellence. Bringing together and systematically learning from thought leaders from these two communities and from the IDx community represented a catalytic first step in conceptualizing an LHS ecosystem for diagnostic excellence. These interviews taught us not only that none of the three communities alone can do the work at hand but also that cocreating a sociotechnical infrastructure is required to realize economies of scale and scope that will underpin the envisioned transformation. However, these lessons only just begin to illuminate our path forward. The work ahead requires collaboration among thought leaders from these three communities as well as patient and health care stakeholders. To plot the path forward and advance on the shared journey, these individuals and organizations will need to work together to map out what needs to be done, where components already exist that can be readily adapted to support it, and what must be built anew to fill the existing gaps. Doing so promises to advance our health system forward in realizing the big, hairy, audacious goal (BHAG) of achieving diagnostic excellence to improve the health of all members of society.

## 6 CONFLICT OF INTEREST

The authors have no conflict of interest to disclose.

## Supporting information

Supporting info item
