# Supplementary material for: Understanding the roles of three academic communities in a prospective learning health ecosystem for diagnostic excellence
Source: Learn Health Syst. 2019 Dec 2;4(1):e210204. doi: 10.1002/lrh2.10204 (PMC6971119; doi:10.1002/lrh2.10204)
Supplement: Supplementary file 1 — Supporting info item [file LRH2-4-e210204-s001.zip › LRH21020-supp-0001-4Semi-structured interview_AI_ver9.29.docx]

Thank you for taking time today to speak with me. I work for a diagnostic improvement project funded by the Gordon and Betty Moore Foundation. Specifically this project will explore how three communities of researchers—those currently studying diagnosis, those focused on Learning Health Systems, and those developing machine learning and AI techniques applicable to medical diagnosis—might come together to address this goal. With this interview, I’m hoping to learn about your own research and explore your views about this potential collaboration.

The important ideas you provide today will contribute directly to a White Paper we will be preparing for the Foundation later this fall.

The interview has two parts. In the first, we’ll focus on machine learning and related methods, particularly as they apply to medical diagnosis; in the second we’ll talk a bit about Learning Health Systems.

**Before we begin,** do I have permission to record our conversation to aid my notetaking?

*Part I: Machine Learning and AI Research*

1. I’d like to start by getting a sense of your personal journey to applying machine learning and AI to health-related problems. How did your interest in these problems develop?
2. Tell me about your specific research and how your work to date has contributed to solving health-related problems.
3. Looking beyond your own research, what have been the major accomplishments of machine learning and AI applied to medical diagnosis in the past 5 years?
4. Looking forward, what are the most important questions surrounding the application of machine learning techniques to the improvement of medical diagnosis?
5. What progress to do you expect to see in the next 5-10 years? What will emerge as the most important methods contributing to this progress?
6. As your field begins to identify methods to improve diagnosis, the challenge remains to translate that knowledge into practice. What are your ideas for how this might be done? Can you point me to good examples of how this translation is happening now?
   [*Cue:* How would or do you collaborate across disciplines into clinical practice? What connections, resources, mechanisms, or infrastructure are missing to make that happen?]

*Part II: Learning Health Systems*

1. In 2007 the U.S. Institute of Medicine advanced the concept of the Learning Health System. What does this term mean to you?
   1. *(If interviewee seems reasonably knowledgeable about the LHS)*
      Based your understanding of the term, in what ways might approaches associated with the Learning Health System be applied to improve diagnosis?
   2. Are you personally involved in any work that relates to the Learning Health System?
   3. What do you see as the relationship between the Learning Health System and machine learning?

***Conclusion:***  Thank you for your time and your excellent thoughts. I hope we can contact you again as this work proceeds. If you think of anything you wanted to add, feel free to e-mail me.
