# Supplementary material for: Understanding the roles of three academic communities in a prospective learning health ecosystem for diagnostic excellence
Source: Learn Health Syst. 2019 Dec 2;4(1):e210204. doi: 10.1002/lrh2.10204 (PMC6971119; doi:10.1002/lrh2.10204)
Supplement: Supplementary file 1 — Supporting info item [file LRH2-4-e210204-s001.zip › LRH21020-supp-0002-Semi-structured interview_Diagnosis_9-29.docx]

Thank you for taking time today to speak with me. I work for a diagnostic improvement project funded by the Gordon and Betty Moore Foundation. Specifically this project will explore how three communities of researchers—those currently studying diagnosis, those focused on Learning Health Systems, and those developing machine learning techniques—might come together to address this goal. With this interview, I’m hoping to learn about your own research and explore your views about this potential collaboration.

The important ideas you provide today will contribute directly to a White Paper we will be preparing for the Foundation later this fall.

The interview has two parts. In the first, we’ll focus on diagnostic research; in the second we’ll talk a bit about Learning Health Systems and machine learning.

**Before we begin,** do I have permission to record our conversation to aid my notetaking?

*Part I: Research on Diagnosis*

1. I’d like to start by getting a sense of your personal journey to studying medical diagnosis. How did your interest in this problem develop?
2. Tell me about your research and how your work has contributed to defining the diagnostic error problem and finding or adopting solutions.
3. Looking beyond your own research, what have been the major accomplishments in research on improving diagnosis in the past 10 years?
4. What are the most important questions going forward?
5. Thinking of potential collaborations among researchers in medical diagnosis, what mechanisms, resources, or infrastructure that are not currently in place would—if added—catalyze progress in the field?
6. As the field begins to identify methods to improve diagnosis, the challenge remains to translate that knowledge into practice. What are your ideas for how this might be done? Can you point me to good examples of how this translation is happening now?
   [*Cue:* Describe how you would prioritize and engage stakeholders--HCOs, providers, patients, payers, HIT?]

*Part II: The Other Communities in this Potential Collaboration*

1. In 2007 the U.S. Institute of Medicine advanced the concept of the Learning Health System. What does this term mean to you?
   1. *(If interviewee seems reasonably knowledgeable about the LHS)*
      Based your understanding of the term, in what ways might approaches associated with the Learning Health System be applied to improve diagnosis?
      [*Cue:* How can an LHS support your mission of improving diagnosis? Where do you perceive limitations?]
   2. Are you personally involved in any work that relates to the Learning Health System?
2. Tell me about how you perceive the potential for ‘big data’ and ‘machine learning’ in improving medical diagnosis.
   [*Cue:* how does this potential make you feel excited, uncertain, or uncomfortable?]

***Conclusion:***  Thank you for your time and your excellent thoughts. I hope we can contact you again as this work proceeds. If you think of anything you wanted to add, feel free to e-mail me.
