# Supplementary material for: Understanding the roles of three academic communities in a prospective learning health ecosystem for diagnostic excellence
Source: Learn Health Syst. 2019 Dec 2;4(1):e210204. doi: 10.1002/lrh2.10204 (PMC6971119; doi:10.1002/lrh2.10204)
Supplement: Supplementary file 1 — Supporting info item [file LRH2-4-e210204-s001.zip › LRH21020-supp-0003-Semi-structured interview_LHS_ver10.4.docx]

Thank you for taking time today to speak with me. I work for a diagnostic improvement project funded by the Gordon and Betty Moore Foundation. Specifically this project will explore how three communities of researchers—those currently studying diagnosis, those focused on Learning Health Systems, and those developing machine learning and AI techniques applicable to medical diagnosis—might come together to address this goal. With this interview, I’m hoping to learn about your own research and explore your views about this potential collaboration.

The important ideas you provide today will contribute directly to a White Paper we will be preparing for the Foundation later this fall.

The interview has two parts. In the first, we’ll focus on the Learning Health System. In the second, we’ll talk more about how the LHS can be applied as a framework toward the goal of improving medical diagnosis and how that might connect to machine learning research.

**Before we begin,** do I have permission to record our conversation to aid my notetaking?

*Part I: Learning Health Systems*

1. I’d like to start by getting a sense of your personal journey to working on Learning Health Systems. How did your interest in these approaches develop?
2. Briefly tell me about your specific research and how your work to date has contributed to the development and implementation of LHSs.
3. Looking beyond your own research, what have been the major accomplishments toward implementing an LHS? Cam you point me to good examples of real-world implementations of learning health systems?
4. Looking forward, what are the most important questions in the development of an LHS?

*Part II: The Learning Health Systems and Interdisciplinary Collaboration*

1. Where do you see the interaction of an LHS framework with mechanisms built specifically to improve medical diagnosis?
   1. As a framework, the LHS necessitates that knowledge is translated into practice. More specifically, how do you see LHS approaches translating knowledge into practice to improve diagnosis?
2. Researchers in diagnostic error focus on cognitive and system processes that can be intervened among three groups of core stakeholders: healthcare organizations, providers, or patients (and to a lesser extent, payers and HIT). How would you prioritize and engage these stakeholders in building an LHS infrastructure?
3. Describe your vision for how LHS researchers and advocates can better tap into the work of our colleagues who work in machine learning techniques and AI? What can we learn from them now and in the future?
   1. In what ways can machine learning and AI work to improve diagnosis?

***Conclusion:***  Thank you for your time and your excellent thoughts. I hope we can contact you again as this work proceeds. If you think of anything you wanted to add, feel free to e-mail me.
